# Correlates of longitudinal leukocyte telomere length in the Costa Rican Longevity Study of Healthy Aging (CRELES): On the importance of DNA collection and storage procedures

**DOI:** 10.1371/journal.pone.0223766

**Published:** 2019-10-11

**Authors:** Luis Rosero-Bixby, David H. Rehkopf, William H. Dow, Jue Lin, Elissa S. Epel, Jorge Azofeifa, Alejandro Leal

**Affiliations:** 1 Centro Centroamericano de Población, Universidad de Costa Rica, San Jose, Costa Rica; 2 School of Medicine, Division of Primary Care and Population Health, Stanford University, Stanford, CA, United States of America; 3 Health Policy and Management, University of California Berkeley, Berkeley, CA, United States of America; 4 Biochemistry and Biophysics, University of California San Francisco, San Francisco, CA, United States of America; 5 Psychiatry, University of California San Francisco, San Francisco, CA, United States of America; 6 Escuela de Biología, Universidad de Costa Rica, San Jose, Costa Rica; University of Newcastle, UNITED KINGDOM

## Abstract

The objective is to identify cofactors of leukocyte telomere length (LTL) in a Latin American population, specifically the association of LTL with 36 socio-demographic, early childhood, and health characteristics, as well as with DNA sample collection and storage procedures. The analysis is based on longitudinal information from a subsample of 1,261 individuals aged 60+ years at baseline from the Costa Rican Study of Longevity and Healthy Aging (CRELES): a nationally representative sample of elderly population. Random effects regression models for panel data were used to estimate the associations with LTL and its longitudinal changes. Sample collection procedures and DNA refrigerator storage time were strongly associated with LTL: telomeres are longer in blood collected in October-December, in DNA extracted from <1-year-old blood cells, and in DNA stored at 4°C for longer periods of time up to five years. The data confirmed that telomeres are shorter at older ages, as well as among males, and diabetic individuals, whereas telomeres are longer in the high-longevity Nicoya region. Most health, biomarkers, and early childhood indicators did not show significant associations with LTL. Longitudinal LTL variation over approximately two years was mainly associated with baseline LTL levels, as found in other studies. Our findings suggest that if there is unavoidable variability in season of sample collection and DNA storage time, these factors should be controlled for in all demographic and epidemiologic studies of LTL. However, due to unobserved components of measurement variation, statistical control may be inadequate as compared to standardization of data collection procedures.

## Introduction

Telomeres are canonical repetitions of non-coding nucleotides and associated protective proteins at the end of chromosomes. By protecting against the degradation of the coding regions of DNA, telomeres: 1) prevent the deletion of coding DNA sequence that would impact transcription and other processes, 2) prevent end to end chromosome fusions that would otherwise occur, and 3) prevent the cell from leaving the cell cycle and becoming senescent. Assays for telomere length from human DNA samples taken from blood indicate the net sum of multiple factors: the degree of telomere length replenishment in stem and other precursor cells by telomerase, telomeric DNA attrition caused by oxidative and other damage to telomeric DNA, as well as replication history occurring in the course of progressive cell divisions within the aggregate of circulating lymphocytes of that individual.

Given that direct experimental manipulation of leukocyte telomere length in humans is not currently possible, knowledge of the underlying causes and consequences of telomere length have been investigated primarily through observational studies, most of which have taken place in Western Europe and the United States. In terms of environmental and behavioral correlates of leukocyte telomere length (LTL), the most consistent associations are with depression and severe stress [1, 2] and in some cases with measures of socioeconomic status that are established earlier in life [3, 4]. There are also fairly consistent findings with tobacco use and physical activity, including some evidence from experimental studies [5, 6]. Some genetic studies have found associations of telomere length and cardiovascular disease risk [7] and Alzheimer's dementia [8]. Newer literature has suggested that leukocyte telomere length may be most useful both on its own [9] and as part of composite measures of earlier life biological markers of the underlying aging process [10, 11].

There are four substantial limitations to the literature that are particularly salient given the reliance on observational studies. Most studies of telomere length have been done in North American and European populations, and there are an increasing number of studies in Asia [12–15] and other parts of the world [3, 16–21]. However, there are almost no studies of telomere length in populations from Central and South American. It is unknown whether findings from other regions are generalizable to these populations. While one smaller study has been done on a targeted population [22], we are aware of no other studies of LTL from nationally representative samples from Central and South America.

A second limitation of the literature is that most studies have focused on correlations with one or two factors at a time, resulting in a potentially biased literature in terms of the lack of reporting of null findings [23]. While a recent study using U.S. data has in part addressed this with physical and environmental measures and their relation to leukocyte telomere length [24], the Costa Rican Study of Longevity and Healthy Aging (CRELES) has a number of factors that were not available in the U.S. data, including early life exposures, which may be important correlates of telomere length [25].

A further limitation of the current literature is that most of the nationally representative population samples with telomere length are based on only one measurement of telomere length. Multiple measures help to reduce the overall measurement error, which may be driving much of the observed changes [26, 27]. Panels also allow examining how change in telomere length is related to environmental, behavioral and health factors. There is literature suggesting that *change* in leukocyte telomere length may be a more relevant metric than static levels [28–30]. For time-varying factors, multiple observations allow estimating models that identify relationships that are free of confounding effects from non-time varying factors, observed or unobserved.

Finally, there has been little work done to examine how attributes of blood sample collection and DNA storage time may affect assayed LTL. A natural quasi-experiment took place in the collection of CRELES data with substantial variability in blood collection season, and in the storage-time of blood cells and DNA, as well as in two LTL-assay batches. We are thus able to use this information to examine whether any of these characteristics had an impact on the measured LTL. In prior work in the CRELES data we reported that month of blood draw was associated with LTL [31], and we attempt to replicate this finding in our new larger sample.

The studied population––elderly Costa Ricans—is known for having a life expectancy that is higher than expected given the middle level of income of this Central American country of five million inhabitants. It is mostly a mestizo population with mean admixture proportions of 46% European, 33% Native American, 13% African and 9% Chinese [32]. According to the 2011 census, only 2% of the national population self-identify as indigenous people and 1% as afro-descendent.

Addressing the aforementioned limitations in the literature, this analysis had two research goals: 1) To determine how LTL correlates with characteristics of elderly individuals in the domains of demographic, socioeconomic status (SES), health conditions, biomarkers, and early childhood conditions, in a Latin American population, where almost no studies have been conducted. 2) To identify research procedures confounding measurement of LTL, a critical issue, especially for longitudinal studies.

## Methods

The study sample was derived from the CRELES cohort, a longitudinal study of a nationally representative sample of 2,827 residents of Costa Rica aged 60 and older at baseline in 2004–6, with oversampling of the oldest old. The study sample also included information from a CRELES-complementary 100% sample of 91 quasi-centenarians (age 95 and above) from the Nicoya region. Longitudinal data came from a second wave of interviews conducted in 2006–8. All CRELES data, examinations, and specimens were taken in the homes of participants. Certified phlebotomists collected fasting blood early in the morning (7–9 am) by venipuncture in three 5ml tubes, one with anticoagulant (ETDA). Plasma and the cell fraction were separated by centrifugation within the next 2–4 hours after collection (9–11 am) and stored in 2ml vials at –40°C. Trained field workers conducted simple physical exams, including two blood pressure, anthropometric, and three hand grip measurements along with a structured interview of more than one hour. Details about the sampling, field, and laboratory procedures are reported elsewhere [33].

The current LTL analysis was conducted in a nested subsample of 1,261 CRELES participants, 968 of them with two observations close to two-years apart and thus amenable to longitudinal analyses. The number of studied LTL observations is thus 2,229. The subsample included all CRELES participants from the high longevity Nicoya region (N = 333, 234 with two observations) and a randomly selected, age-stratified, 38% subsample of CRELES participants from regions other than Nicoya. *S1 Fig* shows a flowchart with the numbers of participants and outcomes in the different stages of this longitudinal survey.

### DNA extraction and LTL measurement

Technicians, in laboratories of the University of Costa Rica, extracted DNAs from the cell fraction after Hermann and Frischauf [34], briefly, the first step is cell lysis and proteinase K digestion, next a phenol-chloroform extraction (tris-buffered phenol/ tris-buffered phenol, chloroform: isoamyl alcohol 24:17/ sodium acetate-isopropanol precipitation/ pellet washing with ethanol 70%) and dissolved in TE buffer. The absorption ratio A260/A280 was 1.72 on average (0.64 s. d.) The extracted DNA samples, with a mean concentration of 106 ng/ul (50 s. d.) were stored at 4°C for periods varying from 0 to 9 years until the LTL assay (12% <1 year, 44% 2–3 years, 7% 4–5 years, 29% 6–7 years, and 8% 8–9 years). A 0.8% agarose gels analysis was run in 2015 to assess integrity of the DNA used to measure telomere length in a systematic selection of 111 DNA samples stratified by years of DNA storage and LTL assay lot. *S1 Document* describe the methods and results of this analysis that concluded there were no apparent DNA degradation.

The Blackburn laboratory at the University of California, San Francisco carried on the LTL assay using quantitative polymerase chain reaction (Q-PCR) to determine the relative ratio of telomere to a single-copy gene (T/S ratio), in this case human beta-globin. Each DNA sample was assayed three times and T and S values were averaged to obtain the T/S ratio. The average inter-assay coefficient of variability was 0.037 (0.033 s. d.) Because of budget constrains, LTL assays were conducted in two lots, with the first lot run in 2010 and the second in 2014. A validation of the results in the two lots was conducted in 29 DNA samples randomly selected from the first lot, which were re-assayed in the second. The correlation coefficient between the two measures of LTL in this subsample was a satisfactory 0.94. However, a bias was detected in the comparison: T/S ratios re-assayed in 2014 were 0.07 longer on average than the original T/S ratios from the 2010 assay. The 95% confidence interval of this mean difference was 0.05–0.09.

### Cofactors

We *a priori* selected 36 possible cofactors of LTL within the domains of measurement procedures, demographic and socioeconomic status (SES), health outcomes, biomarkers, and early child conditions as follows:

1. *Measurement procedures*: years DNA stored at 4 degrees C, whether DNA was extracted from blood cells that were fresh or stored <12 months, season when blood was collected, and lot of the LTL assay (2010 or 2014). Note that season of blood collection and storage times were quasi-random as a result of fieldwork scheduling decisions, as well as due to our random selection of which samples were to be assayed at each time point.
2. *Demographic and SES*: age, sex, thanatological age (time to death), residence in the Nicoya region, if widow, if living alone, education attainment (number of approved years), and income in last month (if married, it is the couple’s average.)
3. *Health outcomes*: self-reported health (international, 1–5 scale of bad health), cancer diagnosis, diabetes diagnosis, taking high-blood pressure medicine, disability scale (need of help in 14 activities of daily life), cognition impairment scale (15-item, version of the Mini-Mental State Examination [35]), and a geriatric depression scale (15-item short form of the Yesavage scale [36]).
4. *Biomarkers*: systolic and diastolic blood pressure (BP), body mass index (BMI), handgrip strength (sex-adjusted by adding 10.8 kg to females), total/HDL cholesterol ratio, triglycerides, C-reactive protein (CRP), glycated hemoglobin (HbA1c), Serum Creatinine (CrS, an indicator of kidney disease), and Dehydroepiandrosterone sulfate (DHEAS). Details about these biomarkers in CRELES have been reported elsewhere [37].
5. *Childhood conditions*: knee height (indicator of nutrition in uterus and infancy), self-reported general health scale (1 = excellent to 4 = poor), had malaria, had asthma, and a general economic hardships scale based on: wearing shoes, sleeping in own bed, having a bathroom and electricity in the house, and being poor.

Table 1 lists the outcome, predictors and measurement factors along with their unit of measurement, weighted average and sampling standard error. The mean age of the studied population is 71.6 years (range 60 to 110 years), about 13% are less than three years from death and 12% from 3–5 years to death, 46% are males, 8% reside in the Nicoya region, 21% are widows, and 10% are living alone.

**Table 1:** Descriptive statistics of the 40 variables in the study.

	Variables	Units	Mean	(S. Err.)	Valid N	Missing
	Telomere length T/S ratio		0.932	(.007)	2,229	0
*Measurement factors*						
	Assay lot 2010	Binary 0–1	0.334	(.019)	2,229	0
	Oct-Dec blood draw	Binary 0–1	0.312	(.015)	2,229	0
	DNA storage time	N. years	5.054	(.086)	2,229	0
	<1-year-old blood cells	Binary 0–1	0.444	(.016)	2,229	0
*Demographic and SES*						
	Exact age	Years	71.399	(.288)	2,229	0
	Deceased in < 3 yrs	Binary 0–1	0.102	(.012)	2,229	0
	Deceased in 3–5 yrs	Binary 0–1	0.117	(.011)	2,229	0
	Sex = male	Binary 0–1	0.459	(.021)	2,229	0
	Nicoya region	Binary 0–1	0.076	(.007)	2,229	0
	Widow	Binary 0–1	0.208	(.014)	2,226	3
	Living alone	Binary 0–1	0.108	(.011)	2,210	19
	Education years	Years	5.474	(.203)	2,229	0
	Monthly income	100,000 Colon	2.274	(.202)	2,201	28
*Health*						
	Self reported poor health	Scale 1–5	3.248	(.037)	2,225	4
	Smoker	Binary 0–1	0.089	(.012)	2,229	0
	Cancer diagnosed	Binary 0–1	0.048	(.008)	2,209	20
	Diabetes diagnosed	Binary 0–1	0.235	(.017)	2,209	20
	Taking BP medicine	Binary 0–1	0.458	(.019)	2,229	0
	ADLs disability	Scale 0–100	16.303	(.803)	2,227	2
	Cognition impairment	Scale 0–100	11.298	(.390)	2,226	3
	Depression symptoms	Scale 0–100	17.285	(.801)	1,477	752
*Biomarkers*						
	Systolic BP	mmHg	144.004	(.831)	2,208	21
	Diastolic BP	mmHg	83.074	(.428)	2,208	21
	BMI	Kg/m2	26.697	(.215)	2,204	25
	Handgrip strength	kg	32.469	(.276)	1,980	249
	Total/HDL cholesterol	ratio	5.059	(.055)	2,211	18
	Triglycerides	mg/dl	164.385	(3.322)	2,209	20
	CRP	mg/l	5.451	(.254)	2,185	44
	HbA1c	percent	5.990	(.051)	2,193	36
	Serum creatinine	mg/dl	0.983	(.016)	2,213	16
	DHEAS	ug/dl	52.851	(1.836)	2,197	32
*Early childhood*						
	Knee height	cm	49.419	(.142)	2,226	3
	Childhood poor health	Scale 1–4	2.229	(.044)	1,550	679
	Childhood malaria	Binary 0–1	0.091	(.012)	1,550	679
	Childhood asthma	Binary 0–1	0.110	(.015)	1,544	685
	Childhood hardship	Scale 0–1	0.483	(.015)	1,556	673

N is the number of observations. Sampling weights and robust standard errors (correction for clustering) were used.

Table 1 also shows the number of missing observations. Most indicators have zero, or very few, missing values. Participants who required a proxy respondent (approximately 30%) lack the information for the geriatric depression scale and the retrospective information on childhood conditions.

### Statistical analysis

All analyses used the CRELES sampling weights modified to account for subsampling fractions. To determine LTL cofactors we fitted to the panel random-effects (RE) multivariate regression models with the telomere T/S ratio as the dependent variable. We preliminarily fitted separate regressions for each explanatory factor to estimate “base models” or crude associations with LTL. These base-model regressions included controls for age, sex, Nicoya residence, and measurement factors. Then, we fitted to the panel a full model with all factors as explanatory variables and, to gauge the confounding effect of measurement factors, we also fitted a non-adjusted full model without these factors in the regression. To further determine whether longitudinal LTL changes are associated with baseline characteristics, we estimated ordinary least squares (OLS) multivariate regression models on the annual LTL change as the dependent variable and baseline levels as explanatory variables. Two of these models were logistic regressions explaining the probability of LTL attrition and the probability of LTL elongation.

In most analyses, the statistically significance cutoff level was set at a more demanding P < 0.01, instead of the conventional 0.05, to avoid potential type-I errors from multiple testing the association of LTL with 36 factors.

Since a substantial portion of each sample was missing data for at least one predictor, in the multivariate regressions, we followed standard practices of multiple imputation to maximize the use of available information [38]. We created five imputed datasets using regression techniques to fill in missing values; the predictors comprised all of the variables in this analysis plus several auxiliary variables that were correlated with non-response (e.g., interviewed by proxy, wave of data, sampling weight). Then, we estimated the model for each imputed dataset and combined the five sets of estimates using Rubin’s rules [39]. All measures of fit and predictive ability were calculated for each dataset and then averaged following the same rules.

### Ethics statement

The Ethical Science Committee of the University of Costa Rica granted human subjects approval to CRELES (VI-763-CEC-23-04). All participants granted written informed consent by means of their signature.

## Results

**Fig 1:**
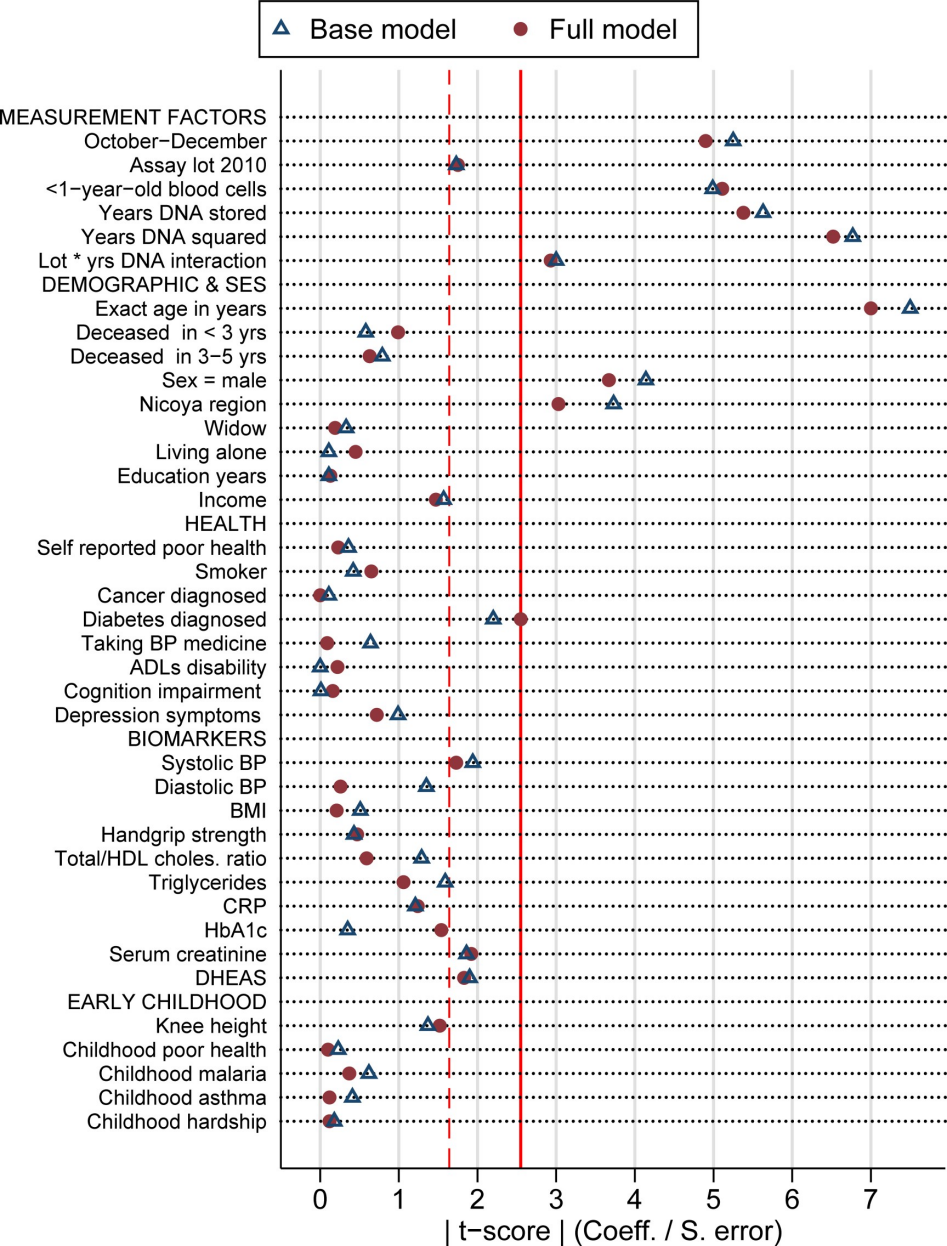
Significance of the association with LTL of 39 explanatory variables in three regression models, as measured by the absolute value of the t-ratio.

Fig 1 summarizes the associations of 36 factors (39 indicators) with LTL measured as T/S ratio according to the base and the full models by showing the absolute *t-scores* (ratio of the regression coefficient to its standard error) as indicator of the strength of the association with LTL. A line at t-ratio = 2.58 identifies the cut-off for an estimate to be statistically significantly different than zero with a probability of <1% type-I error. Another line at t-ratio = 1.64 shows significance at a less demanding <10% error (which could erroneously identify 4 out of our about 40 variables as statistically significant by chance alone.)

Fig 1 suggests no indicators of health, biomarkers or early childhood are statistically significantly associated with LTL at P < 0.01, with the exception of being diabetic in the full model. In contrast, all measurement procedures are significantly associated with telomere length; i.e. quarter when blood was drawn, whether DNA was extracted from <1-year-old blood cells, the duration of DNA storage, and the LTL assay lot (in interaction with storage time). In the group of demographic and SES characteristics, three indicators show a significant association (P < 0.01) with LTL—age, sex, and Nicoya region. Thanatological age (or number of years to death) is not associated to LTL, neither are widowhood, living alone, education, or income.

Marginally non-significant (P < 0.10) associations with LTL are detected for systolic blood pressure, serum creatinine, and DHEAS levels.

Tables 2 and 3 show the direction and magnitude of the regression model estimates of the amount of change in the telomere T/S ratio associated to one unit of change in each of the 39 explanatory variables. Table 2 shows the associations of LTL with DNA storage and sample collection variables and Table 3 the associations of LTL with the four groups of substantive factors. We focus on estimates obtained with the full-model results. The crude, or “base models”, estimates from fitting separate regression models to each variable provide information about associations with LTL that could be hidden by multicolinearity and over-controlling in the full, multivariate model.

**Table 2:** Regression coefficients of measurement procedures explaining LTL.

Measurement factors	Base models			Full model		
Assay lot 2010	-.0603	(.0349)	+	-.0630	(.0360)	+
Oct-Dec blood draw	.0384	(.0073)	**	.0363	(.0074)	**
DNA from <1-year-old blood cells	.0405	(.0081)	**	.0429	(.0084)	**
Years DNA stored	.0657	(.0117)	**	.0652	(.0121)	**
Years DNA squared	-.0064	(.0009)	**	-.0064	(.0010)	**
Lot * storage interaction	-.0239	(.0080)	**	-.0243	(.0083)	**

Estimates with Random-effects (RE) models for panel data using multiple imputation to account for missing values of covariates.

Standard errors in parentheses. Significance:

** P< 0.01

* P<0.05

+ P<0.10

The “base model” estimates come from fitting separate regression models to each variable with controls for age, sex and Nicoya as well as for the remaining measurement factors

The “full model" also included in the regression all substantive factors shown in Table 1.

**Table 3:** Regression coefficients of substantive cofactors explaining LTL.

Substantive explanatory factors	Base models			Full model Adjusted			Full model Non-adjusted		
*Demographic & SES*									
Exact age in years	-.0044	(.0004)	**	-.0042	(.0006)	**	-.0052	(.0007)	**
Deceased in < 3 yrs.	.0070	(.0121)		.0128	(.0129)		.0036	(.0142)	
Deceased in 3–5 yrs.	-.0084	(.0106)		-.0069	(.0110)		-.0141	(.0121)	
Sex = male	-.0356	(.0086)	**	-.0471	(.0128)	**	-.0530	(.0142)	**
Nicoya region	.0381	(.0102)	**	.0337	(.0111)	**	-.0051	(.0120)	
Widow	.0032	(.0097)		.0019	(.0098)		-.0031	(.0108)	
Living alone	-.0013	(.0119)		-.0054	(.0121)		.0011	(.0135)	
Education years	-.0001	(.0012)		.0002	(.0014)		.0023	(.0015)	
Income	-.0014	(.0009)		-.0014	(.0010)		-.0016	(.0011)	
*Health*									
Reported poor health	.0013	(.0036)		.0009	(.0040)		.0006	(.0044)	
Smoker	-.0068	(.0162)		-.0107	(.0165)		-.0063	(.0182)	
Cancer diagnosed	.0020	(.0177)		.0000	(.0177)		.0039	(.0196)	
Diabetes diagnosed	-.0233	(.0106)	*	-.0301	(.0119)	**	-.0264	(.0131)	*
Taking BP medicine	-.0051	(.0079)		-.0007	(.0083)		.0056	(.0091)	
ADLs disability	.0000	(.0002)		.0000	(.0002)		.0000	(.0002)	
Cognition impairment	.0000	(.0003)		-.0001	(.0004)		.0001	(.0004)	
Depression symptoms	.0002	(.0002)		.0001	(.0002)		.0002	(.0002)	
*Biomarkers*									
Systolic BP	.0003	(.0001)	*	.0004	(.0002)	+	.0004	(.0002)	+
Diastolic BP	.0004	(.0003)		-.0001	(.0004)		-.0002	(.0005)	
BMI	-.0004	(.0008)		-.0002	(.0008)		-.0005	(.0009)	
Handgrip strength	.0003	(.0007)		.0003	(.0007)		.0005	(.0008)	
Total/HDL Choles. ratio	-.0030	(.0024)		-.0016	(.0027)		.0028	(.0029)	
Triglycerides	-.0001	(.0000)		-.0001	(.0001)		-.0002	(.0001)	*
CRP	.0006	(.0005)		.0006	(.0005)		.0004	(.0005)	
HbA1c	.0012	(.0034)		.0058	(.0038)		.0125	(.0040)	**
Serum creatinine	-.0206	(.0111)	+	-.0218	(.0114)	+	-.0057	(.0122)	
DHEAS	.0002	(.0001)	+	.0002	(.0001)	+	.0003	(.0001)	*
*Early childhood*									
Knee height	.0023	(.0017)		.0028	(.0018)		.0031	(.0020)	
Childhood poor health	-.0013	(.0056)		-.0006	(.0060)		.0000	(.0062)	
Childhood malaria	-.0097	(.0157)		-.0049	(.0131)		-.0145	(.0154)	
Childhood asthma	.0072	(.0174)		.0018	(.0144)		.0172	(.0159)	
Childhood hardship	.0032	(.0176)		.0021	(.0175)		.0001	(.0186)	
Constant				.9425	(.1134)	**	1.0437	(.1209)	**

Estimates with Random effects (RE) models for panel data using multiple imputation to account for missing values of covariates.

Standard errors in parentheses. Significance:

** P< 0.01

* P<0.05

+ P<0.10

The “base models” are separate regression models for each variable with controls for measurement factors, age, sex, and Nicoya.

The “full models” included in the regression all 32 explanatory variables in this Table; the “adjusted model” also included in the regression the measuring factors of Table 2.

Results are essentially similar with both the base and full models.

### LTL and measurement procedures

Blood samples drawn in the October-December quarter (final months of the Costa Rican rainy season) are associated with 0.4 longer telomere T/S ratios (Table 2). DNA extracted from a <1-year-old blood cell is associated with longer telomere by 0.04 T/S ratio. DNA storage time and assay lot are related to LTL in a complex, non-linear pattern as shown by the significant effects of a quadratic term of storage time and the interaction term of storage and assay lot (other statistical interactions that were non-significant in preliminary analyzes were not included in the models). The estimates in Table 2 suggest that longer periods of DNA storage result in longer telomeres (by 0.07 T/S ratio in the first three years), but this effect diminishes over time and reverses after about five years of storage (the T/S ratio is 0.09 shorter in the ninth storage year, compared to the fifth year). The LTL assays conducted in 2014 tend to be 0.16 T/S ratio longer than the 2010 assays in DNA samples with four storage years (there were no DNA samples with less than four storage years in the 2014 lot). S2 *Document* shows a plot of the complex combined effects of storage time and assay lot.

Note that the estimated “lot effect” of 0.16 T/S ratio in DNA with 4 years of storage is substantially higher than the previously mentioned raw effect of 0.07 found in the replication set of 29 DNA samples. This discrepancy probably comes from uncontrolled confounding biases in the raw assessment, especially related to different DNA storage times in the two lots.

### Substantive factors associated to LTL

Table 3 shows the estimates for the substantive factors, including two versions of the full model: one controlling the confounding effects of measurement procedures—our optimal estimates, and a suboptimal model with non-adjusted estimates. According to our optimal estimates, the telomere T/S ratio diminishes by 0.04 with ten years of age; males have 0.05 shorter T/S ratios; adults from the Nicoya region have 0.03 longer telomeres, and diabetic individuals have 0.03 shorter telomeres. All these associations are highly significant at P<0.01.

There are also three estimates marginally non-significant (P <0.10): an effect of 0.004 higher telomere T/S ratio with each 10 mmHg increase in systolic BP; 0.002 higher T/S ratio with one ug/L increase in DHEAS, and 0.02 shorter telomere with a unit increase in serum creatinine.

How different would be the associations with LTL if measurement procedures were not controlled for in our regression models? The non-adjusted estimates in the last columns of Table 3 show a big picture not that different than that from the adjusted estimates: most factors under analysis (23 out of 32) are not significantly associated with telomere length, whereas five out of nine significant associations take place in both models. This similarity occurs because variations in DNA collection and storage were to some extent randomly distributed. Results significantly different occur in just four variables: the non-adjusted model suppressed the associations with LTL of residence in Nicoya and kidney disease, and gave spurious significant associations of telomeres with triglycerides and HbA1c.

In spite of the above general assessment of a lack of confounding effect of measurement procedures, a Hausman test comparing the two models suggests systematic differences in the estimates of the two models (*S3 Document*). Interestingly, the standard errors in Table 3 are systematically larger (10% larger on average) in the non-adjusted model, meaning that controlling for measurement procedures results in more efficient estimates.

### The choice of RE models rather than fixed-effects (FE) models

Our methodological decision of using RE regression models to fit our panel data was actually made after observing in preliminary analyses that FE models were substantially less efficient (larger standard errors), as warned by the literature [40], and after checking that a Hausman test does not reject the more efficient RE-model in favor of fixed effects. The comparison of FE and RE estimates and the resulting Hausman test are shown as support information *S3 Document*.

### Sex and age stratification

To test whether results in Tables 2 and 3 differ by sex or by age, we re-estimated the full-adjusted model adding statistical interactions between each variable in the model and sex and dichotomized age (being 80 or more years old). Prior observations have noted that telomere length declines with age up to around age 80, and increases with age after the age of 80 [16]. No interaction with sex was statistically significant, while two LTL associations were different at older ages. First, LTL shortens by 0.05 T/S with ten years of age among people aged 60 to 79 years, whereas it shortens by only 0.02 among people aged 80 or more. Second, LTL is not associated with systolic blood pressure among younger individuals, while it shortens by 0.007 T/S ratio with reductions of 10 mmHg in systolic BP at older ages. Notably, the effects of measurement factors did not statistically differ by sex or age groups. *S1 Table* shows the full results from the age-stratified model.

### LTL dynamics

In the panel of 968 individuals with two observations, the correlation coefficient between the two LTL measurements was a moderate R = 0.57, despite of the short interval of less than two years between waves. This suggests considerable volatility in our LTL measure, either because LTL has a substantial natural intra-individual variability or because of measurement error.

To compute the longitudinal variation in LTL we first normalized all T/S ratios to hypothetical values that would have been obtained if DNA extraction and LTL assay were conducted shortly after blood samples drawing (i.e. on <1-year-old blood cells and zero DNA storage time) as well as using the same lab procedures as in the 2014 assay lot. With the regression coefficients of the full-model shown in Table 2 we obtained the normalized T/S ratios (*S2 Document* shows the normalization equations and a figure with the density distributions before and after normalization).

The change over time in LTL distributed normally around a central value of zero (*S2 Document*), meaning that the number of individuals with shortening telomeres was approximately the same as the number with enlarging telomeres. With the unadjusted LTL indicator, LTL was shorter over time in 48% of the sample; with the normalized LTL indicator, telomere attrition occurred in 53% of the sample.

Many changes observed in telomere length were small changes close to zero. Taking |0.1| annual change in the normalized T/S ratio as cut-off (0.1 is approximately one standard deviation of the T/S ratio), we identified the occurrence of telomere shortening in 12% of the sample and, lengthening in 11%, with thus 77% of individuals with no or little telomere change between sample times, which were on average 1.8 years apart.

Table 4 shows the results of three multivariate regression models addressing the question of how baseline factors impact longitudinal change in LTL. In order to reduce the number of multiple comparisons in our analysis, these models include only the 14 explanatory variables that showed some significant association with LTL in some of our preliminary analyses. The dependent variable is the annual change in the normalized T/S ratio.

**Table 4:** Regression coefficients explaining prospective LTL change with baseline factors.

Explanatory variables at baseline	OLS regression on LTL change&			Logistic regressions on:					
				LTL attrition&			LTL elongation&		
Baseline LTL	-.3107	(.0156)	**	8.7026	(.7893)	**	-6.1275	(.8346)	**
Oct.-Dec. draw	-.0235	(.0064)	**	.3075	(.2499)		-.4194	(.2976)	
Exact age in years	-.0020	(.0004)	**	.0391	(.0151)	**	-.0655	(.0152)	**
Deceased in 3–5 years	-.0079	(.0074)		.1874	(.3120)		-.2931	(.3316)	
Sex = male	-.0106	(.0078)		.4666	(.3279)		-.4972	(.3183)	
Nicoya region	.0100	(.0069)		-.0463	(.2784)		.3741	(.2824)	
Income	-.0003	(.0009)		.0404	(.0307)		-.0035	(.0370)	
Diabetes diagnosed	-.0152	(.0083)	+	-.5257	(.3926)		-.6738	(.3724)	+
Cognition impairment	.0003	(.0003)		-.0178	(.0114)		-.0051	(.0123)	
Systolic BP	.0001	(.0001)		.0023	(.0048)		-.0016	(.0047)	
Grip hand strength	-.0002	(.0005)		-.0061	(.0209)		-.0162	(.0204)	
CRP	-.0002	(.0004)		-.0094	(.0180)		-.0117	(.0175)	
HbA1c	.0017	(.0028)		.1475	(.1059)		-.0388	(.1328)	
Serum creatinine	-.0092	(.0088)		.2396	(.3048)		.3582	(.3418)	
DHEAS	.0001	(.0001)		-.0019	(.0032)		-.0023	(.0031)	
Knee height	-.0007	(.0012)		-.0163	(.0477)		-.0004	(.0470)	
Constant	.4453	(.0678)	**	-13.3614	(2.8844)	**	9.0125	(2.8481)	**

&LTL change is a continuous metric of the difference between follow-up LTL and baseline LTL. LTL attrition is a dichotomous indicator of individuals who had 0.1 lower LTL at the second measurement, and LTL elongation is a dichotomous indicator of individuals who had 0.1 higher LTL at the second measurement.

Significance:

** P< 0.01

* P<0.05

+ P<0.10

Note that models in Table 4 included baseline LTL among the explanatory variables. This baseline LTL level is, precisely, the strongest predictor of LTL change: individuals with longer telomeres are more likely to experience telomere shortening and, conversely, individuals with shorter telomeres are more likely to experience LTL elongation. While baseline LTL level is highly correlated with LTL change, this may be due to correction of measurement error and/or “regression to the mean” corrections of extreme low or high baseline values, rather than there being an actual mechanistic or causal relationship between baseline LTL and change over time.

The longer baseline telomeres of blood drawn in October-December are associated with significantly smaller (or negative) rate of prospective change, meaning that individuals with this characteristic are more likely to experience LTL attrition and less likely to experience LTL elongation.

Older individuals, males, and diabetics also show negative rates of LTL change of some statistical significance, which means that LTL attrition is more likely among them.

## Discussion

Our primary findings support a new descriptive understanding of the relationships of LTL with socio-demographic and health factors among elderly people in the Latin American context of Costa Rica. Among nine socio-demographic factors, we find that age, sex, and residence in Nicoya are associated with LTL. Among 18 health and biomarker predictors, we found associations with diabetes and, marginally non-significant, with systolic blood pressure and serum creatinine. We found no associations with five early childhood measures. We also found associations between prospective change in LTL and baseline LTL, quarter of blood collection, age, and being diabetic.

Our findings also offer new insights into sample collection and DNA storage attributes that impact assayed LTL. In CRELES, T/S ratios tend to be significantly higher in DNA extracted from <1-year-old blood cells or if blood was drawn at the end of the rainy season (October to December); they increase with the storage time of DNA until about four years declining thereafter; and they vary significantly between the two LTL-assay batches in these data. In considering our findings on DNA storage time, it is important to emphasize that DNA was stored at 4 degrees C; storage at -20 C or -70 C may have different impacts on assayed LTL. In spite of the apparent effect of DNA storage duration, a high correlation (r = 0.94) between two measures taken four years apart on the same DNA samples suggest that rank order comparisons may be preferred over comparisons of actual values. With respect to the findings that telomere length was longer during the rainy season, our leading hypothesis is that there are seasonal difference in constituent cell types by season, perhaps due to infection prevalence. Prior work has shown that telomere length differs by cell type [41]. While we do not have data on cell types, future work on expanded samples of CRELES using DNA methylation data will allow us to estimate cell types and test this hypothesis [42]. The relatively strong magnitude of association between these factors and LTL suggests that sample collection variation is not a minor factor, but is strong enough to potentially spuriously produce a large degree of bias if not addressed through sample design and analysis procedures.

Prior literature addressing the issue of LTL measurement validity cautions about the noise from intra- and inter-laboratory technical variations or from the use of different LTL assay methods, as well as from different DNA extraction methods [43–45]. Prior work suggested that storage of DNA evaluated for up to two and a half years at 4 degrees C did not have detrimental impacts on the yield of the DNA or the quality of the DNA [46]. By contrast, another study showed that DNA samples at 1 ng/uL were very weakly correlated to their original results when stored at 4 C and only moderately correlated when stored at -30 C, whereas DNA at 25 ng/uL maintained strong correlations to the original results after 6 months at both 4 C and -30 C [47]. However, T/S ratios measured after 6 months of storage were systematically lower than the original values, possibly due to DNA degradation. Other work has showed the PCR-based assays of telomere results can differ depending on DNA extraction method, with column based DNA less likely to find an association with cancer risk as compared to phenol/chloroform (which we used) and salting out [45]. While it is unclear how this may be related to the impacts of DNA storage time in terms of how it is affecting the results of the PCR based assay, our results build on these prior findings suggest how critical the handling and storage of DNA is for the assay.

A separate possibility is that DNA degradation was the cause of LTL variations in our sample: DNA stocks were not stored under completely sterile conditions, and resulting bacterial and fungal nucleases could potentially alter telomere length. Residual chemicals and impurities from the extraction procedure can also have a cumulative effect over time on the qPCR reactions. Oxidation of DNA during storage may also alter the telomeric and single copy gene PCR reactions differently to cause apparent higher T/S ratios. To further investigate DNA degradation as a possible cause of LTL correlations with DNA storage time, we ran 0.8% agarose gels on 111 samples systematically selected in order to have representation of the different storage times. With the exception of 5 samples that didn’t show a band, the remaining 106 samples all looked intact with no indication of degradation, thus the underlying source of LTL correlation with storage time remains a question for future analysis (details in *S1 Document*).

Three socio-demographic characteristics were clearly associated with LTL in our study: age, sex, and residence in the high-longevity Nicoya region. The CRELES data confirm that LTL is shorter with age—a dynamic expected on theoretical bases [48, 49] and confirmed empirically by dozens of studies [50]. The T/S ratio decreased by 0.05 every 10 years of age among individuals aged 60–79 years and by 0.02 among those aged 80 years or more. As in a majority of published studies, LTL was shorter among males—by 0.05 T/S ratio in our study. The longer LTL of Nicoyans confirmed an earlier report based on a smaller number of observations [51]. The lack of association between SES indicators and LTL in our data contrasts with findings in the United States, where higher education is associated with longer telomere length [3]. A different association with SES indicators in the Costa Rican context, however, is not completely unexpected, as factors associated with SES differ between the U.S. and Costa Rica [52], and the overall associations between SES and mortality are much weaker in Costa Rica [53].

A striking result of this study is the mostly lack of association between 18 health risk factors and biomarkers with LTL. The exceptions were: individuals with diagnosed diabetes, who showed 0.03 shorter T/S, and the marginally non-significant associations with LTL of Systolic BP, serum creatinine, and DHEAS. There are some similarities to these results in prior studies of biomarkers and LTL in the United States, especially regarding the lack of association with LTL of triglycerides, glucose, HbA1C, and diastolic BP [23, 24]. However here are also discrepancies, including the lack of association in the U.S. with systolic BP and serum creatinine and the existence of associations with BMI and CRP, absent in our data.

Indicators of early childhood hardship were not associated with shorter LTL. We did not however have indicators of psychological stress in childhood, which has been the most consistent findings with telomere length thus far in the literature [25, 54].

Our data confirms findings from other longitudinal studies that the most important determinant of longitudinal variation in LTL is its baseline level, in a pattern that some literature interpret as suggestions that some of the observed variations, especially those after extreme low or high baseline values, might be just “regression to the mean” corrections or measurement error corrections [55–57]. Individuals with longer LTL are more likely to experience telomere shortening in a follow up visit and, conversely, individuals with shorter LTL are more likely to experience LTL elongation. About 11% of studied individuals experienced LTL enlargement (increase in more than one standard deviation) which usually is attributed to measurement error, especially in short follow up periods such as the less than two years of the CRELES panel [58, 59]. LTL shortening occurred in 12% of individuals and this dynamics was more likely to occur among older individuals and among diabetics.

A limitation to our findings is that DNA was taken from blood, and thus our results are specific to the mean telomere length in circulating nucleated leukocytes, and telomere length differs depending on cell type. It is also critical to acknowledge the limitation that we could not control for different leukocyte composition, which is important since telomere length is also specific to white blood cell type [41]. Even controlling for crude measures of cell type has shown to reduce coefficients of association[23].

The short follow up period of less than two years is also an important limitation of our longitudinal data. Samples taken farther apart—say, 5 or 10 years—might have yielded more meaningful changes in telomere length than those found in this article.

As shown by our analysis of “measurement factors,” the heterogeneities in DNA extraction (<1-year-old vs. older blood cells), not storing DNA at freezing temperatures, and assays made in two separate, four-year apart lots are also limitations that this study tried to control with statistical instruments.

However, two findings give us reasonable evidence that there was not substantial enough measurement error in the assays to nullify our results. First, that we found similar associations to the prior literature in LTL associations with demographic factors like age and sex. Secondly, there were not substantially different findings when not adjusting for the measurement error. Thus even as the environmental conditions seem to have resulted in some changes in LTL, they were not substantial enough to dramatically change the inferences in our study.

In summary, our findings suggest that there are stronger associations of LTL with demographic factors than with health and biomarker outcomes, and that some of these relationships differ from those observed in the U.S., Europe and Asia. These findings suggest that LTL may be driven by different factors within different contexts, even as the sex and age associations appear to be universal. A true test of this, however, will need to involve a more careful consideration of inter-assay variation of LTL and make these comparisons directly. Our findings suggest that all future demographic and epidemiological work with LTL should attempt to minimize variation in DNA storage time and sample collection procedures and avoid any systematic variation with respect to sample characteristics. However, if this is not possible due to the use of archival samples, these characteristics should be statistically controlled for in analyses.

Given the effects of DNA storage time and assay lots on telomere length measurement, we recommend that future studies with longitudinal telomere length measurements should engage in rigorous quality control as well as repeat testing. A substantial subset of samples drawn earlier would ideally be assayed once after minimal storage, and then frozen and re-assayed later in the same batch as each wave of subsequent samples. This would allow for estimation and control of both storage time and assay lot effects. We opted in this article for a sub-optimal solution of statistically adjusting TL measurement, which might be biased by unobserved confounding factors.

## Supporting information

S1 Data Stata (version 15) dataset with CRELES variables used in this analysis (N = 2,229). (DTA)

S1 Document Agarose gels analysis to assess DNA degradation. (PDF)

S2 Document Supporting information regarding normalization of LTL measurements. (PDF)

S3 Document Hausman tests comparing pairs of regression models. (PDF)

S1 Fig Flowchart of CRELES data used in LTL analyses. (PDF)

S1 Table Regression coefficients of measurement and substantive factors explaining LTL stratified by two large age groups. (PDF)
