# Supplementary material for: Correlates of longitudinal leukocyte telomere length in the Costa Rican Longevity Study of Healthy Aging (CRELES): On the importance of DNA collection and storage procedures
Source: PLoS One. 2019 Oct 11;14(10):e0223766. doi: 10.1371/journal.pone.0223766 (PMC6788698; doi:10.1371/journal.pone.0223766)
Supplement: S1 Document — (PDF) [file pone.0223766.s002.pdf]

S1 Document. Agarose gels analysis to assess DNA degradation.

A 0.8% agarose gels analysis on 111 DNA samples was conducted to determine possible DNA degradation. The DNA samples were systematically selected after stratification of all studied samples in 11 groups resulting of combining the LTL-study lot (2010 or 2014) and the number of years of DNA storage. The molecular size marker (Bioline HyperLadderTM 1 kb) was run on the same gel. The lab personnel who run the agarose gels were blind to the DNA storage time information. The agarose gels were run in September 2015.

S2 Table 1 shows the characteristics of the DNA samples in this analysis.

S2 Fig 1 shows the pictures resulting from the agarose gels with a number identifying each of the DNA samples in S2 Table 1.

The Fig shows that, with the exception of 5 samples that didn't show a band, all the remaining 106 samples have a single discrete band well above the 10kb marker that is consistent with DNA being intact and with no apparent degradation. This result does not support the hypothesis that DNA degradation is the underlying source of LTL correlation with DNA and blood cells storage time .

S1 Document Table 1. Characteristics of the 111 DNA samples in agarose gels.

idsujeto	wave	T/S ratio	YR stored blood cells	YR stored DNA til LTL assay	YR stored DNA til agarose gel	LTL lot	OD260/ OD280	ID in gel image	Gel appearance
3610	1	0.771	0	5	10	2010	.	1	intact
3	1	0.776	0	5	10	2010	1.71	2	intact
99	1	0.720	0	5	10	2010	1.47	3	No DNA
49	1	0.849	0	5	10	2010	1.86	4	intact
3635	1	0.831	0	5	10	2010	1.79	5	intact
1395	1	1.209	0	5	10	2010	1.82	6	intact
3567	1	1.040	0	5	10	2010	1.77	7	intact
4161	1	0.764	0	5	10	2010	1.83	8	intact
4092	1	0.798	0	5	10	2010	1.81	9	intact
1589	1	0.935	0	5	10	2010	1.82	10	intact
1558	1	0.834	0	5	10	2010	1.84	11	intact
5059	1	0.745	0	3	8	2010	1.84	12	intact
5357	1	0.829	0	3	8	2010	1.87	13	intact
5599	1	0.726	0	3	8	2010	1.85	14	intact
5183	1	0.609	0	3	8	2010	1.9	15	intact
2832	2	0.745	0	2	7	2010	1.91	16	intact
4181	2	0.792	0	2	7	2010	1.9	17	intact
9066	1	0.745	0	3	8	2010	1.89	18	intact
9071	1	0.823	0	3	8	2010	1.9	19	intact
9156	1	0.838	0	3	8	2010	1.85	20	No DNA
9183	1	0.844	0	3	8	2010	1.52	21	intact
1203	1	0.590	4	0	5	2010	1.87	22	intact
9217	1	0.904	0	3	8	2010	1.77	23	intact
1880	2	1.000	0	2	7	2010	1.86	24	intact
2563	2	0.678	0	2	7	2010	1.83	25	intact
2093	2	0.528	0	2	7	2010	1.9	26	intact
1481	2	0.784	3	0	5	2010	1.79	27	intact
1485	2	0.947	3	0	5	2010	1.83	28	No DNA
1494	2	0.893	3	0	5	2010	1.87	29	intact
1760	2	0.574	3	0	5	2010	1.88	30	intact
1761	2	0.842	3	0	5	2010	1.75	31	intact
1441	2	0.481	3	0	5	2010	1.84	32	intact
2241	2	0.822	0	2	7	2010	1.86	33	No DNA
1190	1	0.832	4	0	5	2010	1.58	34	intact
1915	2	0.855	0	2	7	2010	1.87	35	intact
1198	1	0.688	4	0	5	2010	1.86	36	intact
3940	2	0.650	3	0	5	2010	1.89	37	intact
1490	2	0.703	3	0	5	2010	1.68	38	intact
1372	2	0.856	0	2	7	2010	1.76	39	intact
1382	2	0.700	0	2	7	2010	1.87	40	intact
1389	2	0.889	0	2	7	2010	1.85	41	intact

idsujeto	wave	T/S ratio	YR stored blood cells	YR stored DNA til LTL assay	YR stored DNA til agarose gel	LTL lot	OD260/ OD280	ID in gel image	Gel appearance
1241	2	0.746	0	3	8	2010	1.87	42	intact
4232	1	0.967	5	0	5	2010	1.82	43	intact
4197	1	0.762	5	0	5	2010	1.84	44	intact
4164	1	1.008	5	0	5	2010	1.89	45	intact
2704	1	0.706	5	0	5	2010	1.86	46	intact
683	1	0.780	4	0	5	2010	1.89	47	intact
1423	2	0.581	3	0	5	2010	1.87	48	intact
1440	2	0.862	3	0	5	2010	1.89	49	intact
2492	1	0.792	5	0	5	2010	1.9	50	intact
2273	1	0.915	4	0	5	2010	1.91	51	intact
2241	1	0.779	4	0	5	2010	1.86	52	intact
2471	1	0.549	4	0	5	2010	1.89	53	intact
2474	1	0.781	4	0	5	2010	1.89	54	intact
1130	1	0.601	4	0	5	2010	1.88	55	intact
2596	1	0.828	5	0	5	2010	1.86	56	intact
2540	1	0.671	5	0	5	2010	1.88	57	intact
302	1	0.757	5	0	5	2010	1.88	58	intact
2453	1	0.674	4	0	5	2010	1.87	59	intact
2178	1	0.573	5	0	5	2010	1.89	60	intact
3354	1	0.745	5	0	5	2010	1.89	61	intact
1659	2	0.752	2	0	5	2010	1.86	62	intact
1725	2	0.704	2	0	5	2010	1.87	63	intact
1413	2	0.602	2	0	5	2010	1.87	64	intact
654	2	0.841	2	0	5	2010	1.88	65	intact
3173	2	0.683	2	0	5	2010	1.85	66	intact
5462	2	0.679	2	0	5	2010	1.67	67	intact
9136	2	0.627	2	0	5	2010	1.9	68	intact
9154	2	0.740	2	0	5	2010	1.89	69	intact
9210	2	0.603	2	0	5	2010	1.9	70	intact
9202	2	0.688	2	0	5	2010	1.91	71	intact
1194	2	0.867	0	6	7	2014	1.84	72	intact
1550	1	0.773	0	9	10	2014	1.91	73	No DNA
1565	1	0.794	0	9	10	2014	1.91	74	intact
159	1	0.751	0	9	10	2014	1.91	75	intact
2224	1	1.053	0	9	10	2014	1.9	76	intact
2268	2	0.742	0	6	7	2014	1.89	77	intact
2280	2	0.967	0	6	7	2014	1.9	78	intact
2297	2	0.989	0	6	7	2014	1.88	79	intact
2335	2	0.785	0	6	7	2014	1.77	80	intact
2517	2	0.98	0	6	7	2014	1.76	81	intact
2595	2	0.854	0	6	7	2014	1.92	82	intact
2615	1	1.861	0	7	8	2014	1.92	83	intact
2619	1	1.262	0	7	8	2014	1.87	84	intact

idsujeto	wave	T/S ratio	YR stored blood cells	YR stored DNA til LTL assay	YR stored DNA til agarose gel	LTL lot	OD260/ OD280	ID in gel image	Gel appearance
2632	1	0.671	0	7	8	2014	1.93	85	intact
2956	1	1.267	0	9	10	2014	1.86	86	intact
3041	1	0.56	0	9	10	2014	1.91	87	intact
3447	2	1.25	0	6	7	2014	1.89	88	intact
3595	1	0.804	0	9	10	2014	1.9	89	intact
3601	1	0.93	0	9	10	2014	1.89	90	intact
3652	2	0.628	0	6	7	2014	1.86	91	intact
392	1	0.772	0	8	9	2014	1.92	92	intact
399	1	0.819	0	8	9	2014	1.93	93	intact
4045	1	0.814	0	8	9	2014	1.88	94	intact
414	1	1.121	0	8	9	2014	1.92	95	intact
415	1	0.849	0	8	9	2014	1.87	96	intact
418	1	0.676	0	9	10	2014	1.9	97	intact
4225	2	0.8	0	6	7	2014	1.93	98	intact
447	1	0.781	0	8	9	2014	1.91	99	intact
452	1	0.777	0	8	9	2014	1.94	100	intact
454	1	0.852	0	8	9	2014	1.94	101	intact
484	1	0.824	0	8	9	2014	1.9	102	intact
5005	1	0.903	0	7	8	2014	1.91	103	intact
501	1	1	0	8	9	2014	1.91	104	intact
5048	1	0.974	0	7	8	2014	1.91	105	intact
5080	1	0.653	0	7	8	2014	1.92	106	intact
51	1	0.917	0	9	10	2014	1.9	107	intact
5205	1	0.841	0	7	8	2014	1.94	108	intact
5221	1	0.947	0	7	8	2014	1.93	109	intact
5355	1	1.104	0	7	8	2014	1.89	110	intact
5492	1	0.745	0	7	8	2014	1.91	111	intact

S1 Document Fig 1. Agarose gels images of 111 DNA samples selected from the CRELES-LTL analysis

Gel # 1

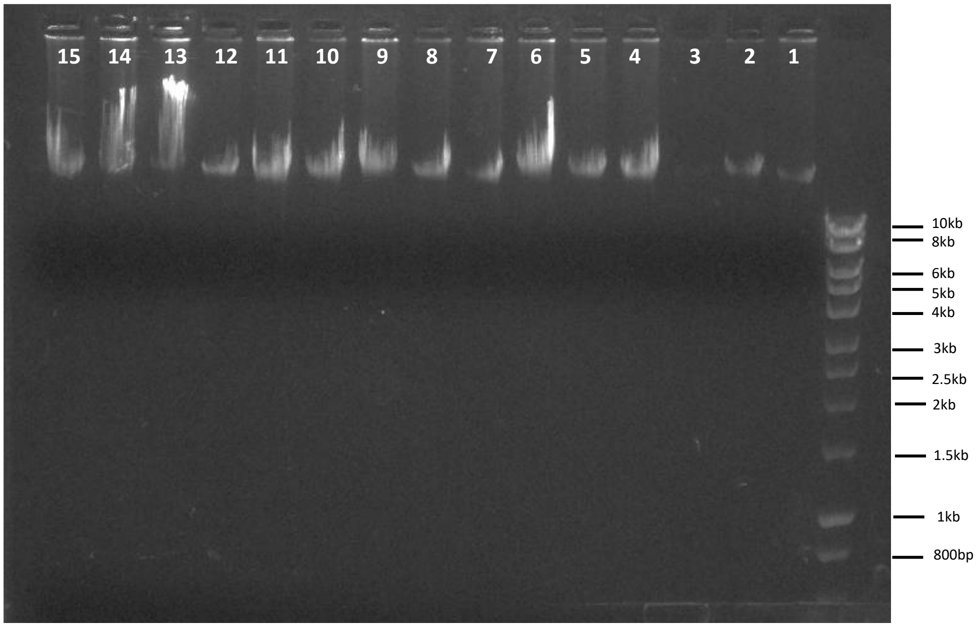

Gel # 2

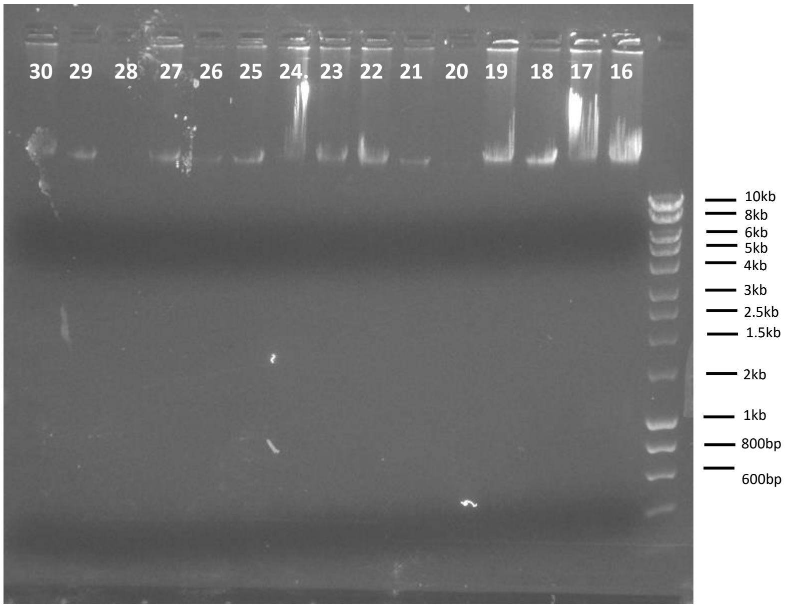

Gel # 3

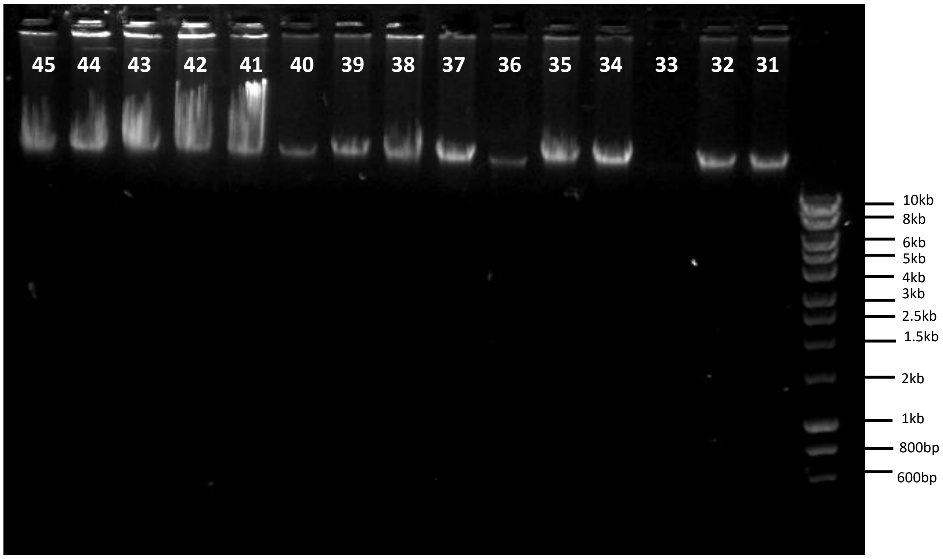
45 44 43 42 41 40 39 38 37 36 35 34 33 32 31
10kb
8kb
6kb
5kb
4kb
3kb
2.5kb
1.5kb
2kb
1kb
800bp
600bp

Gel # 4

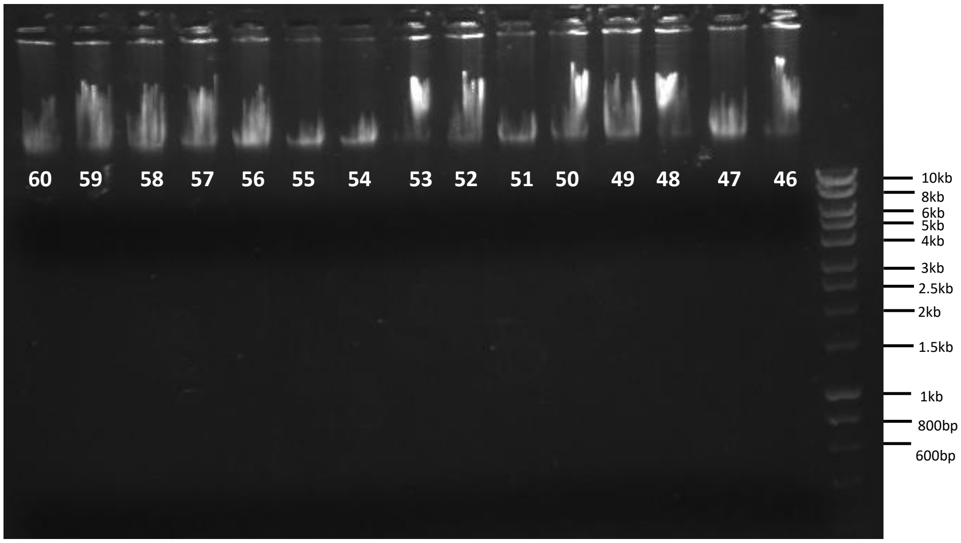
60 59 58 57 56 55 54 53 52 51 50 49 48 47 46
10kb
8kb
6kb
5kb
4kb
3kb
2.5kb
2kb
1.5kb
1kb
800bp
600bp

Gel # 5

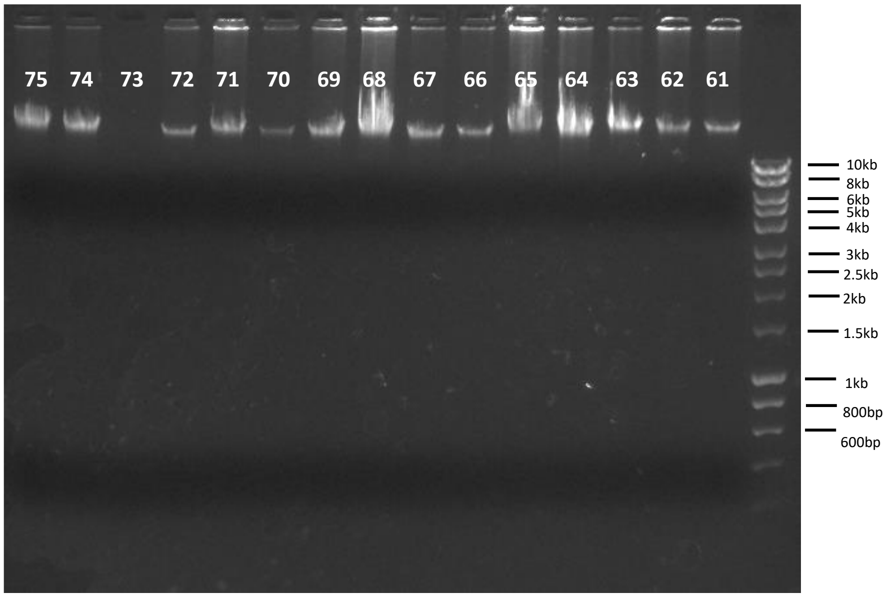
75 74 73 72 71 70 69 68 67 66 65 64 63 62 61
10kb
8kb
6kb
5kb
4kb
3kb
2.5kb
2kb
1.5kb
1kb
800bp
600bp

Gel # 6

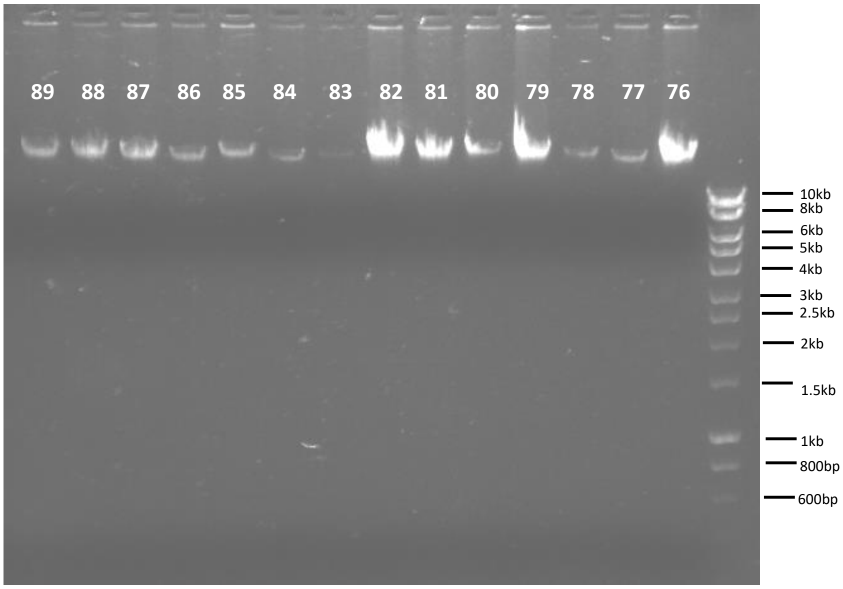
89 88 87 86 85 84 83 82 81 80 79 78 77 76
10kb
8kb
6kb
5kb
4kb
3kb
2.5kb
2kb
1.5kb
1kb
800bp
600bp

Gel # 7

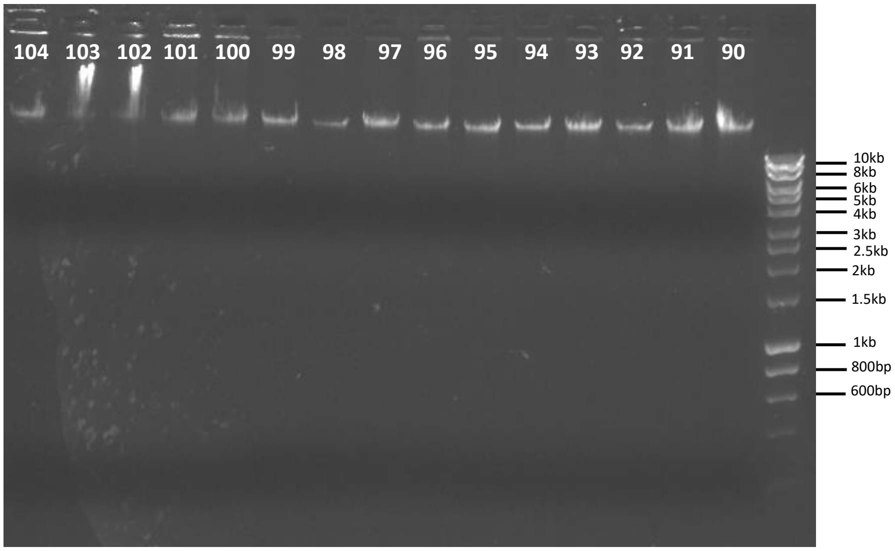
104 103 102 101 100 99 98 97 96 95 94 93 92 91 90
10kb
8kb
6kb
5kb
4kb
3kb
2.5kb
2kb
1.5kb
1kb
800bp
600bp

Gel # 8

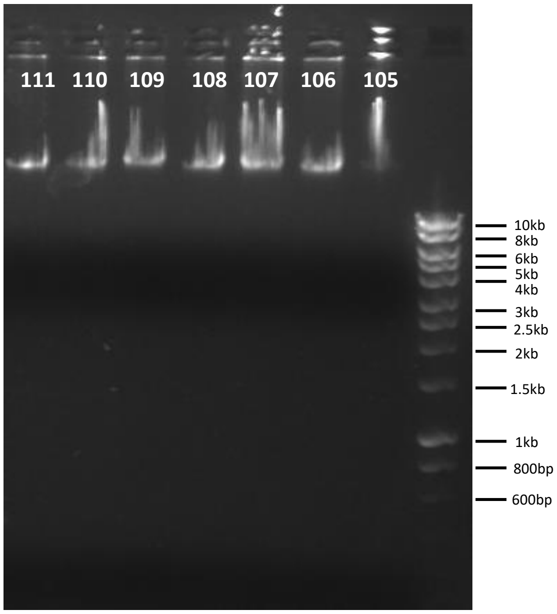
111 110 109 108 107 106 105
10kb
8kb
6kb
5kb
4kb
3kb
2.5kb
2kb
1.5kb
1kb
800bp
600bp
