# Supplementary material for: Correlates of longitudinal leukocyte telomere length in the Costa Rican Longevity Study of Healthy Aging (CRELES): On the importance of DNA collection and storage procedures
Source: PLoS One. 2019 Oct 11;14(10):e0223766. doi: 10.1371/journal.pone.0223766 (PMC6788698; doi:10.1371/journal.pone.0223766)

S2 Document. Supporting information regarding normalization of LTL measurements

Computing a normalized LTL indicator

To make our LTL indicator comparable across all our observations, especially within the same individual over time, we normalized the observed T/S ratios, estimating for each observation the expected T/S ratio if DNA were extracted from <1-year-old blood cells and the LTL assay were conducted immediately (zero storage time of DNA) using the procedures of the 2014 assay. To do so, we used the coefficients from the full model shown in Table 2 of main text to estimate adjusted or normalized T/S ratios with the following equations:

2014 LTL assay:

$$\text{Adj\_T/S} = \text{Obs\_T/S} - (.065\ \text{DNAyrs} - .0064\ \text{DNAyrs}_2) + .043\ \text{oldblood}$$

2010 LTL assay:

$$\text{Adj\_T/S} = \text{Obs\_T/S} - (.041\ \text{DNAyrs} - .0064\ \text{DNAyrs}_2) + .043\ \text{oldblood} + .063$$

Where:

Adj_T/S and Obs_T/S are the adjusted and observed T/S ratios
DNAyrs Time (in exact years) DNA was stored prior to the LTL assay
Oldblood: 1 if blood cells were stored 12 or more months prior to DNA extraction.

The adjustment was derived from multivariate regression analysis including more than 30 indicators of demographic, socioeconomic, health and early childhood characteristics of participants. This analysis shows highly significant effects on the TS ratio of DNA storage time (curvilinear and in interaction with the assay lot) and whether DNA was extracted from <1-year-old blood cells. Fig 1 shows the estimated effects of years of DNA storage. These effects were corrected in the normalized T/S ratio.

Results

Fig 2 shows how the density distribution in the LTL longitudinal variation changed after the normalization, shifting left. The new, normalized variation distribution shows, as expected, that there are more shortening (<0) that enlargement changes (>0).

S2 Document Fig 1. Marginal effects of DNA storage time and assay lot upon LTL

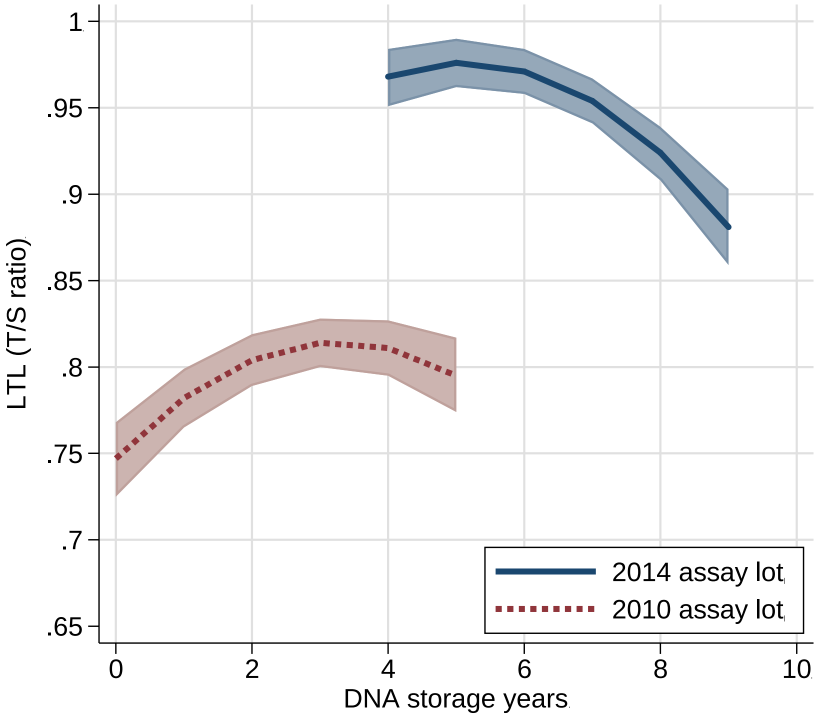

Note that in the 2010 assay lot there were no observations with more than 5 years
of DNA storage and that in the 2014 lot there were no observations with less than 4 years.

S2 Document Fig 2. Density distribution of annual change in LTL

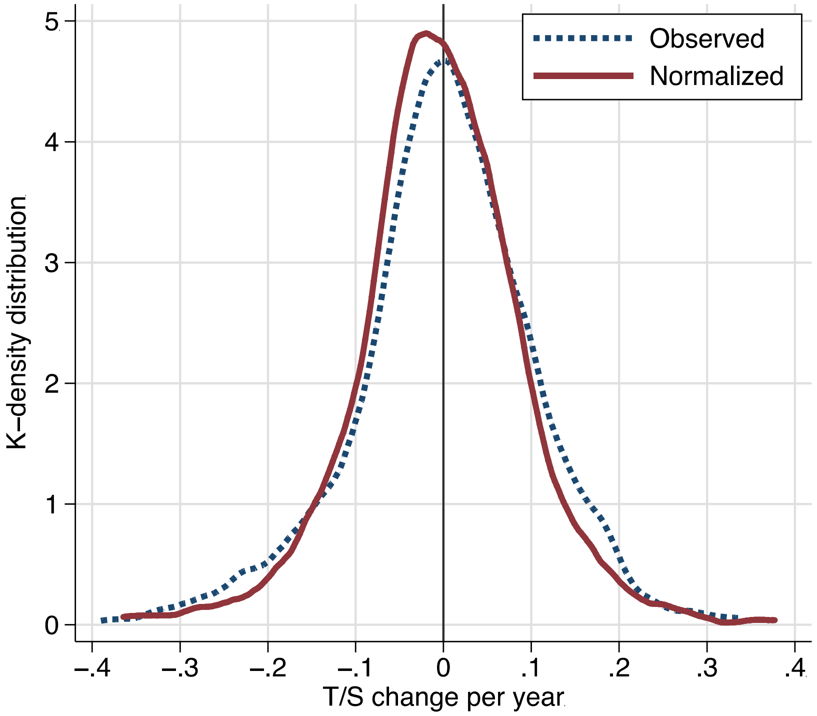

Supplement: S2 Document — (PDF) [file pone.0223766.s003.pdf]
