# Supplementary material for: Correlates of longitudinal leukocyte telomere length in the Costa Rican Longevity Study of Healthy Aging (CRELES): On the importance of DNA collection and storage procedures
Source: PLoS One. 2019 Oct 11;14(10):e0223766. doi: 10.1371/journal.pone.0223766 (PMC6788698; doi:10.1371/journal.pone.0223766)
Supplement: S3 Document — (PDF) [file pone.0223766.s004.pdf]

S3 Document. Hausman tests comparing pairs of regression models

Note: Since computation of the Hausman test was not available in Stata for "multiple imputation" panel regressions, the following tests were conducted without imputations and excluding from the regression six explanatory variables with more than 100 missing values. These exclusions result in coefficients slightly different than those shown in the main text.

S3 Document Table 1. Comparing coefficients of the model adjusted with measure factors with the unadjusted model

	Coefficients		Difference (Adj – Non-adj)	
	Adjusted	Non-adjus.	Diff.	(St. Er.)
Demographic & SES				
Exact age in years	-0.00446	-0.00561	0.00115	(.00011) **
Deceased in < 3 yrs.	0.01262	0.00609	0.00653	(.00156) **
Deceased in 3-5 yrs.	-0.00660	-0.01228	0.00568	(.00112) **
Sex = male	-0.04888	-0.05475	0.00587	(.00126) **
Nicoya region	0.02953	-0.00892	0.03844	(.00291) **
Widow	0.00539	-0.00052	0.00591	(.00103) **
Living alone	-0.00302	0.00485	-0.00787	(.00120) **
Education years	0.00082	0.00270	-0.00188	(.00020) **
Income	-0.00151	-0.00158	0.00007	(.00012)
Health				
Reported poor health	0.00249	0.00318	-0.00069	(.00036) +
Smoker	-0.00926	-0.00659	-0.00268	(.00158) +
Cancer diagnosed	0.00182	0.00328	-0.00147	(.00152)
Diabetes diagnosed	-0.03129	-0.02585	-0.00544	(.00162) **
Taking BP medicine	-0.00383	0.00253	-0.00635	(.00090) **
ADLs disability	-0.00005	-0.00002	-0.00003	(.00002) +
Cognition impairment	0.00002	0.00031	-0.00029	(.00004) **
Biomarkers				
Systolic BP	0.00038	0.00043	-0.00005	(.00002) **
Diastolic BP	0.00002	-0.00019	0.00021	(.00004) **
BMI	-0.00045	-0.00075	0.00030	(.00011) **
Total/HDL Chol. ratio	-0.00197	0.00239	-0.00436	(.00071) **
Triglycerides	-0.00006	-0.00015	0.00009	(.00001) **
CRP	0.00056	0.00020	0.00036	(.00006) **
HbA1c	0.00724	0.01352	-0.00627	(.00114) **
Serum creatinine	-0.02520	-0.01101	-0.01419	(.00298) **
DHEAS	0.00018	0.00025	-0.00007	(.00001) **
Early childhood				
Knee height	0.00340	0.00391	-0.00051	(.00017) **

N = 2014 observations, 1207 individuals. Significance: ** P< 0.01, * P<0.05, + P<0.10

Hausman Test:

Ho: difference in coefficients not systematic

Rejected: chi2(26) = 319.29, Prob>chi2 = 0.0000

S3 Document Table 2. Comparing coefficients of the RE model with the FE model

Explanatory factors	Coefficients		Difference (FE–RE)		
	FE	RE	Diff.	(St. Er.)	
Measurement factors					
Assay lot 2010	-0.12849	-0.06266	-0.06584	(.04246)	+
Oct-Dec blood draw	0.03800	0.03342	0.00458	(.00759)	
DNA from <1-yr-old blood cells	0.07769	0.04617	0.03151	(.01403)	*
Years DNA stored	0.06035	0.06528	-0.00493	(.01042)	
Years DNA squared	-0.00655	-0.00656	0.00001	(.00110)	
Lot * storage interaction	-0.02226	-0.02490	0.00264	(.00902)	
Demographic & SES					
Exact age in years	-0.00665	-0.00446	-0.00219	(.00773)	
Deceased in < 3 yrs.	-0.03166	0.01262	-0.04429	(.02973)	+
Deceased in 3-5 yrs.	-0.01669	-0.00660	-0.01009	(.01907)	
Widow	-0.02862	0.00539	-0.03401	(.02380)	+
Living alone	-0.00830	-0.00302	-0.00528	(.02102)	
Income	-0.00049	-0.00151	0.00103	(.00084)	
Health					
Reported poor health	-0.00120	0.00249	-0.00369	(.00435)	
Smoker	0.03979	-0.00926	0.04906	(.03717)	+
Cancer diagnosed	0.00895	0.00182	0.00714	(.05307)	
Diabetes diagnosed	-0.04077	-0.03129	-0.00948	(.03128)	
Taking BP medicine	-0.01317	-0.00383	-0.00935	(.01505)	
ADLs disability	0.00025	-0.00005	0.00030	(.00034)	
Cognition impairment	-0.00046	0.00002	-0.00048	(.00053)	
Biomarkers					
Systolic BP	0.00006	0.00038	-0.00032	(.00029)	
Diastolic BP	0.00053	0.00002	0.00051	(.00049)	
BMI	0.00171	-0.00045	0.00215	(.00231)	
Total/HDL Chol. ratio	-0.00187	-0.00197	0.00011	(.00400)	
Triglycerides	-0.00011	-0.00006	-0.00005	(.00007)	
CRP	0.00147	0.00056	0.00091	(.00059)	+
HbA1c	0.00723	0.00724	-0.00002	(.00521)	
Serum creatinine	-0.03810	-0.02520	-0.01290	(.02247)	
DHEAS	-0.00009	0.00018	-0.00027	(.00031)	

N = 2014 observations, 1207 individuals. Significance: ** P< 0.01, * P<0.05, + P<0.10
FE = Fixed effects model; RE = Random effects model
Hausman Test:
Ho: difference in coefficients not systematic
Accepted: chi2(26) = 39.32, Prob>chi2 = 0.076
