# Supplementary material for: Correlates of longitudinal leukocyte telomere length in the Costa Rican Longevity Study of Healthy Aging (CRELES): On the importance of DNA collection and storage procedures
Source: PLoS One. 2019 Oct 11;14(10):e0223766. doi: 10.1371/journal.pone.0223766 (PMC6788698; doi:10.1371/journal.pone.0223766)
Supplement: S1 Fig — (PDF) [file pone.0223766.s005.pdf]

S1 Fig. Flowchart of CRELES data used in LTL analyses

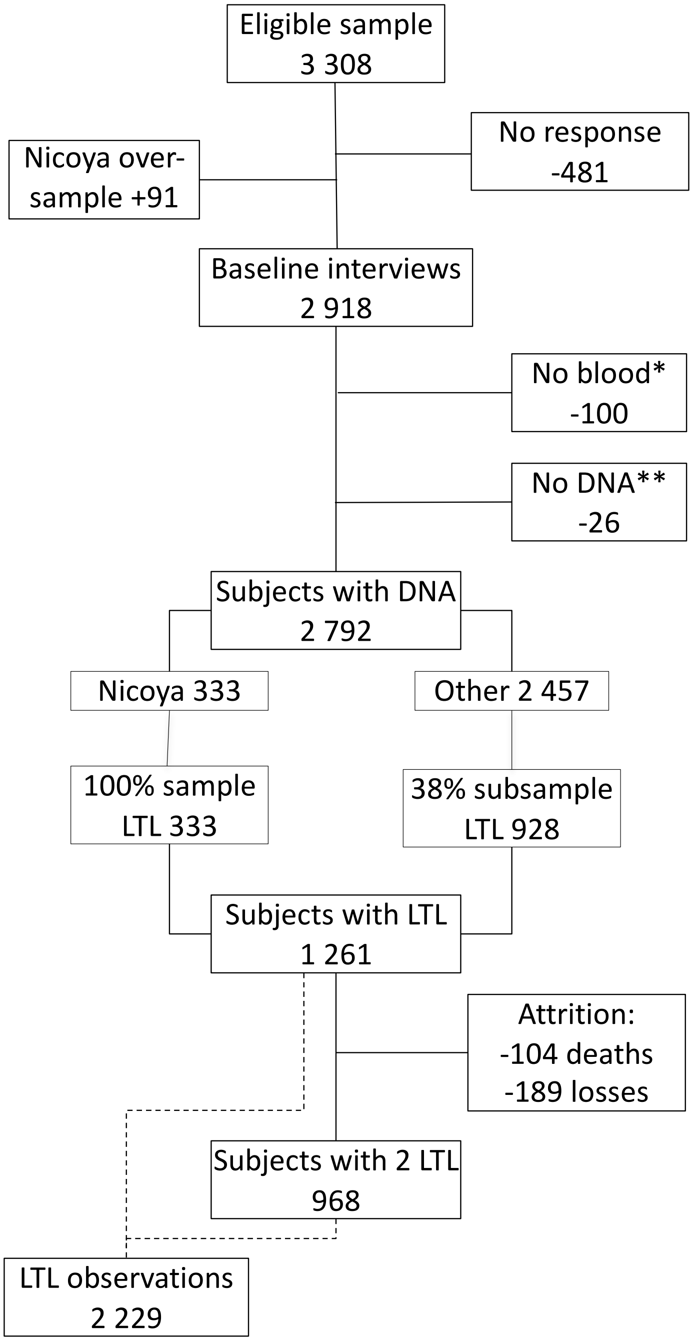

* Rejected venipuncture in both waves of visits

**Individuals with no proper DNA stored
