# Supplementary material for: Correlates of longitudinal leukocyte telomere length in the Costa Rican Longevity Study of Healthy Aging (CRELES): On the importance of DNA collection and storage procedures
Source: PLoS One. 2019 Oct 11;14(10):e0223766. doi: 10.1371/journal.pone.0223766 (PMC6788698; doi:10.1371/journal.pone.0223766)
Supplement: S1 Table — (PDF) [file pone.0223766.s006.pdf]

*S1 Table. Regression coefficients of measurement and substantive factors explaining LTL stratified by two large age groups*

| Explanatory factors | Age 60-79 years Coef. | (95% conf. Interval) | | Age 80+ years Coef. | (95% conf. Interval) | | |
|---|---|---|---|---|---|---|---|
| *Measurement factors* | | | | | | | |
| Oct.-Dec. blood draw | 0.0275 | (0.0072 | 0.0477) | 0.0534 | (0.0327 | 0.0741) | |
| Assay lot 2010 | -0.0887 | -(0.1869 | 0.0094) | -0.0329 | -(0.1351 | 0.0693) | |
| DNA from <1-year-old blood cells | 0.0524 | (0.0289 | 0.0758) | 0.0331 | (0.0097 | 0.0564) | |
| Years DNA stored | 0.0600 | (0.0270 | 0.0930) | 0.0736 | (0.0393 | 0.1079) | |
| Years DNA squared | -0.0063 | -(0.0090 | -0.0036) | -0.0067 | -(0.0095 | -0.0039) | |
| Lot- storage interaction | -0.0216 | -(0.0443 | 0.0011) | -0.0286 | -(0.0522 | -0.0050) | |
| *Scio-demographic* | | | | | | | |
| Exact age in years | -0.0053 | -(0.0076 | -0.0030) | -0.0024 | -(0.0046 | -0.0002) | + |
| Deceased in 3-5 yrs | -0.0041 | -(0.0363 | 0.0281) | -0.0158 | -(0.0381 | 0.0065) | |
| Sex = male | -0.0621 | -(0.0953 | -0.0289) | -0.0343 | -(0.0678 | -0.0008) | |
| Nicoya region | 0.0357 | (0.0033 | 0.0681) | 0.0408 | (0.0139 | 0.0678) | |
| Income (100,000) | -0.0015 | -(0.0039 | 0.0009) | -0.0014 | -(0.0042 | 0.0014) | |
| *Health & biomarkers* | | | | | | | |
| Diabetes diagnosed | -0.0147 | -(0.0445 | 0.0151) | -0.0426 | -(0.0781 | -0.0071) | |
| Cognition impairment | -0.0002 | -(0.0013 | 0.0009) | 0.0000 | -(0.0008 | 0.0009) | |
| Systolic BP (10 units) | 0.0002 | -(0.0041 | 0.0046) | 0.0070 | (0.0029 | 0.0110) | * |
| Grip hand strength | 0.0004 | -(0.0014 | 0.0022) | -0.0002 | -(0.0031 | 0.0026) | |
| CRP | 0.0010 | -(0.0006 | 0.0026) | 0.0007 | -(0.0004 | 0.0019) | |
| HbA1c | 0.0052 | -(0.0038 | 0.0143) | 0.0022 | -(0.0108 | 0.0152) | |
| Serum creatinine | -0.0194 | -(0.0492 | 0.0104) | -0.0207 | -(0.0530 | 0.0115) | |
| DHEAS (10 units) | 0.0024 | -(0.0004 | 0.0051) | 0.0012 | -(0.0023 | 0.0048) | |
| Knee height | 0.0041 | -(0.0008 | 0.0090) | 0.0019 | -(0.0031 | 0.0069) | |
| Constant | 1.0061 | (0.6972 | 1.3150) | 0.7376 | (0.3970 | 1.0782) | |
| Observations | 1223 | | | 1006 | | | |
| Individuals | 695 | | | 619 | | | |

Estimated with "random effect" (RE) regression models on panel data, using multiple imputation to account for missing values of covariates.

Difference between the two age groups significant at: * P<0.05, + P <0.10
